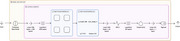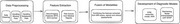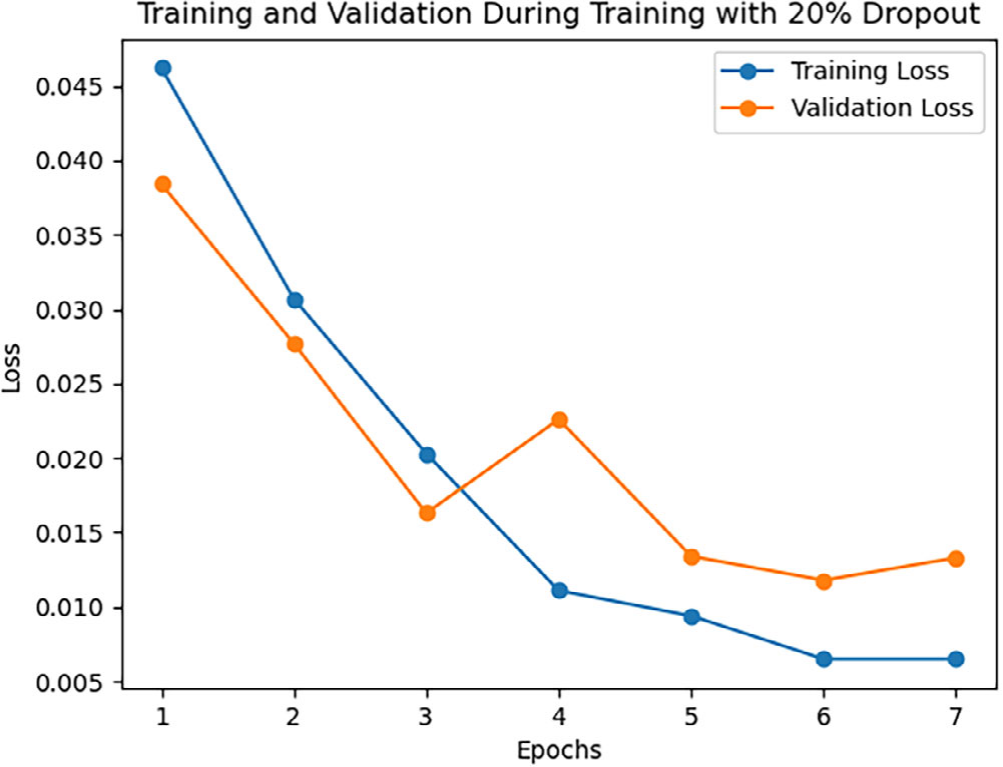# AI‐Driven Pipeline for Analysis and Classification of Neurodegenerative Diseases from Cognitive, Imaging, and Clinical Biomarkers from the CogNID Study

**DOI:** 10.1002/alz70855_106908

**Published:** 2025-12-25

**Authors:** Aditya Purswani, Armaghan Moemeni, Akram A. Hosseini

**Affiliations:** ^1^ University of Nottingham, Nottingham, Nottinghamshire, United Kingdom; ^2^ Nottingham University Hospitals NHS Trust, Queens Medical Center, Nottingham, United Kingdom

## Abstract

**Background:**

Neurodegenerative disorders (ND) like Alzheimer's Dementia (AD) and Fronto‐Temporal Dementia (FTD) pose significant challenges in early diagnosis due to their multifaceted nature and the limitations of traditional diagnostic methods. The Cognitive and Neuroimaging in Neurodegenerative Disorders (CogNID) study provides a comprehensive dataset comprising cognitive evaluations, MRI scans, and clinical biomarkers of 450 patients. This study aims to develop an AI‐driven framework for early detection of dementia by integrating multimodal data.

**Method:**

This study uses a multimodal approach, combining clinical, imaging data, and reports to develop a robust diagnostic pipeline. Radiology reports are pre‐processed using natural language processing (NLP), and domain‐specific contexts, captured by tokenisation and creating embedding using PubMedBERT. BART‐based classifiers are used to generate initial risk context, medical contexts are then examined using clustering and severity rating to generate risk scores for reports. Imaging data include MRI scans which will be analysed by using the FMRIB Software Library (FSL) to extract volumetric features like medial temporal and global cortical atrophy along with including cognitive assessment scores and cerebrospinal fluid (CSF) data which will be fused with the features extracted from scans and reports. Machine Learning methods including ensemble methods, and neural networks will be used for developing models to classify neurodegenerative disorders trained on the fused features. Model evaluation will be performed using metrics such as accuracy, precision, and recall while maintaining model interpretability using techniques like SHAP (Shapley Additive Explanations) to improve trustworthiness.

**Result:**

Preliminary results indicate the successful development of the Medical Risk Analyser (MRA) for processing textual data and generating risk scores. In the loss curve as the number of epochs increases the training and validation accuracy decreases steadily showcasing robust training of the model. Further work is ongoing to extract and integrate features from MRI scans and build classification models by enhancing the computation and increasing the number of training epochs.

**Conclusion:**

This study advances AI applications to support early diagnosis of ND by addressing medical data imbalance, robust model development, and ensuring model interpretability and scalability. This framework can support a relevant and precise diagnosis of dementia, important for further research on patients' diagnoses.